# One Binder to Bind Them All

**DOI:** 10.3390/s16101665

**Published:** 2016-10-10

**Authors:** Oliver Hayden

**Affiliations:** Siemens Healthcare GmbH, Strategy and Innovation, Technology Center, In-Vitro DX & Bioscience, Günther-Scharowsky-Str. 1, 91058 Erlangen, Germany; oliver.hayden@siemens.com; Tel.: +49-9131-7-32804

**Keywords:** sensor, molecularly imprinted polymers, receptor, binder, healthcare, environment, life science, cells, quartz crystal microbalance, lithography, aptamers

## Abstract

High quality binders, such as antibodies, are of critical importance for chemical sensing applications. With synthetic alternatives, such as molecularly imprinted polymers (MIPs), less sensor development time and higher stability of the binder can be achieved. In this feature paper, I will discuss the impact of synthetic binders from an industrial perspective and I will challenge the molecular imprinting community on the next step to leapfrog the current status quo of MIPs for (bio)sensing. Equally important, but often neglected as an effective chemical sensor, is a good match of transducer and MIP coating for a respective application. To demonstrate an application-driven development, a biosensing use case with surface-imprinted layers on piezoacoustic sensors is reported. Depending on the electrode pattern for the transducer, the strong mechanical coupling of the analyte with the MIP layer coated device allows the adoption of the sensitivity from cell mass to cell viability with complete reversibility.

## 1. Introduction

Self-assembly and templating effects are amazing phenomena in nature which have been extensively studied on the molecular to the macroscopic size regime by chemists [1,2]. To take advantage of such effects for chemical sensor development is an attractive strategy but at the same time a challenge as many requirements of both the transducer and sensor layer need to match up for a useful device. In the past two decades we could witness many innovative sensing ideas from the field of molecularly imprinted polymers (MIPs) which appeared to the chemical sensor community as the generally applicable strategy [3]. MIPs were considered as the perfect binder to bind most analytes and do not require large lab investments and resources. However, we have seen that MIP coated sensors have not yet been commercialized and the question we have to ask ourselves is why. New technologies usually show sufficient maturation after some 20 years and thus for many reported MIP sensing technologies one could have expected more impact on real-world applications [4].

## 2. Market Forces Affecting Commercial Success of Chemical Sensors with MIPs

Academia has shown many attractive MIP sensor applications with market potential for small molecule analytes [5,6] and bioanalytes [7] in even complex environments. However, a truly broad industrial applicability of a single MIP platform technology for chemical sensing remains to be seen. For example, a scalable coating technique for biosensing with a MIP synthesis in aqueous conditions remains a significant challenge despite the plethora of polymer chemistry tricks [8]. Furthermore, the chemical sensor market has only a few killer applications with substantial market volume which is attractive enough to develop a supply chain and introduce a novel technology for a highly regulated market such as in vitro diagnostics (IVDs) or environmental sensing in harsh conditions. Strong competition from mature chemical sensor products with natural binders, miniaturized analytical instruments, use of physical sensors, and the increasing use of image analysis, are amongst other additional challenges for a potential market entry for MIP sensors. Considering such market forces, the MIP community needs to identify analytical problems with substantial market need where the chemical sensor layer requirements are in favor of MIPs and the sensing cannot be easily performed with conventional binders. For such applications, attractive pricing could be achieved even in markets with high cost pressure, such as IVD, where improved patient outcome and thus a favorable cost-benefit ratio is the relevant value. MIP sensors could also have a significant impact in targeted applications where capital investment for conventional analytical instrumentation is too high and users are interested in decentralized solutions with low maintenance and servicing, such as for environmental sensing. Last, trends such as the internet of things or continuous self-monitoring requiring long-term stability may have a push effect for widespread usage of chemical sensors as physical sensor information will always have specificity limits.

## 3. Limitations and Opportunities of MIP Coatings for (Bio)Sensing

To briefly introduce the concept of a chemical sensor, I am referring to the very concise definition of Ricco and Crooks [9]: “A successful chemical sensor-based system must respond with application-dependent sensitivity, selectivity, reversibility, speed, and longevity to a desired analyte, while consuming minimal power and volume, not to mention being manufacturable from inexpensive materials using economical batch methods.” Comparing these requirements with reported MIP sensor results, I observe that the majority of reports are still focusing on individual aspects of the sensor, such as the chemical recognition effect. In particular the simultaneous need for high selectivity and complete reversibility is a difficult task to achieve. My general observation with MIPs is that increasing selectivity and affinity causes lower reversibility and response time. This problem usually increases with increasing analyte size, such as proteins or even whole cells. The art of MIP sensing is therefore to identify the right trade-off between analytical performances for the right application.

Naturally, we would like to have one single effortless methodology to fabricate sensor layers which serves all needs for real-world sensing applications. Template-directed polymerization is considered to be such a broadly applicable method. In reality, it is difficult to have all MIP binder properties, such as affinity, specificity, reversibility of the binding process, robustness or ease of fabrication, amongst other at an optimum level. For instance, many MIP sensing applications have trade-offs between response time and sensitivity to ensure complete reversibility which can only be partially compensated with a highly-sensitive transducer. For real-world applications, the understanding of the sensor should not stop at the polymerization of the MIP but needs to cover the transducer properties as well as the whole value chain. When I review MIP sensor applications I still see the modus operandi of taking an available transducer and coating it with a MIP layer. I believe this approach is not sufficient to attract a broad sensor community to apply MIPs and to translate MIP research results into innovative sensor products. In the past years the community has gained a good understanding of the recognition effects using MIPs. Today, we need more effort on demonstrating robust long-term sensor operation in real-life samples, control of drift effects, and complete reversibility. Furthermore, upscaling potential for sensor fabrication is needed and we need attractive business cases to translate MIP sensing. 

For small molecule analytes, MIP coatings have a proven track record for sensing applications usually seizing the bulk of a thin polymeric film to bind low molecular weight analytes. In particular, environmental sensing with small but very specific chemical sensor arrays show readiness for product development [10,11]. Increasing analyte size steric effects make bulk imprinting unfavorable and surface imprinting attractive. At the same time, coating methods, such as spinning or dip coating, applied for small molecule templates are not applicable due to low binding site density and irreversible template binding. I observed that surface moulding of a polymerizing thin film with a soft-lithographic process can be used instead as a rational process for high density MIP sites [12]. The soft-lithographic approach is applicable for any transducer with a flat surface which allows a polymerizing thin film to be spin-coated and to conformally mould the polymer surface with a template coated stamp. However, the generally applicability of soft-lithography for bioimprinting does not, per se, solve the sensing of bulky analytes with surface-imprinted MIP layers. It turns out that the analytical performance of such a MIP sensor depends on the transducer which will be discussed in detail on the use case yeast cell sensing with a MIP coated piezoacoustic sensor. 

## 4. Materials and Methods

**Figure 1:**
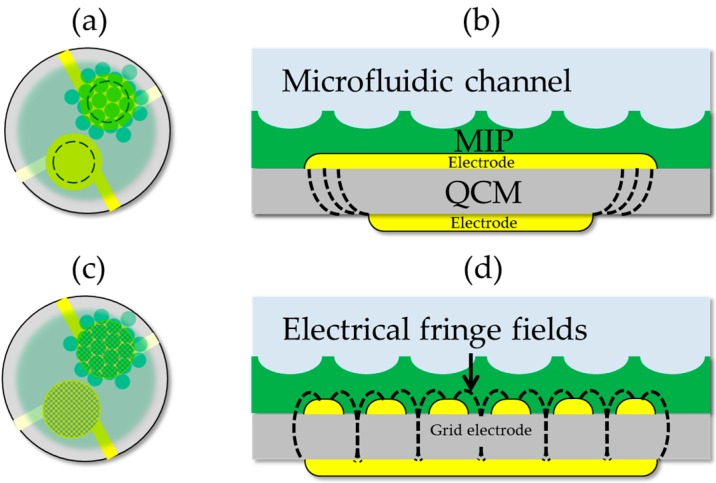
Schematic illustration of the quartz crystal microbalance (QCM) layouts with reference and sensitive electrodes (yellow) covered with a polyurethane thin film (green). The reference electrode is coated with a plane non-imprinted thin film and the sensitive electrode is coated with the surface-imprinted layer. (**a**) Traditional layout with smaller electrodes (broken line) towards the gaseous phase. (**b**) Cross-section of the sensitive layer. With larger electrodes on ground, facing the aqueous phase, the electrical fringe fields (broken lines) are pulled into the crystal. (**c**) QCM layout with a grid electrode facing the aqueous phase. (**d**) Cross-section showing schematic fringe fields at the edges of the grid electrode.

Cell surface imprinting on piezoacoustic devices has been reported elsewhere [12,13]. In brief, screen-printing and low-temperature curing is applied to fabricate AT-cut 10 MHz quartz crystal microbalances (QCMs) with gold electrodes for a sensitive and reference layer. The sensitive surface-imprinted sensor is fabricated with a soft-lithography-related process which allows a reproducible layer of closely packed recognition sites. Pre-polymerized polyurethane layers are spin-coated on the QCMs and surface imprinting is performed by embossing compressed cells as templates onto the polymer thin film. The coated reference electrodes are not surface-imprinted. Both the sensitive and the reference electrodes facing the cell suspension are on ground. The counter electrodes are not immersed and the smaller diameter minimizes the influence of the buffer’s conductivity. The sensor is positioned horizontally at the bottom of a microfluidic flow cell. The yeast cell suspension is continuously transported over the thin film sensor layer and removal of adhered cells is performed at high flow rates where the fluidic drag force exceeds the adhesive forces based on chemical and geometrical recognition of the analytes. To probe dielectric properties of the cells, gold electrodes with a grid structure and ~50% fill factor are used to maximize electrical fringe fields. The fringing field increases the capacitance of the grid electrode and contributes to piezoelectric stiffening [14,15]. The additional liquid lossy electrode is affected by the conductivity and permittivity of the cell suspension and shifts the resonance frequency of the QCM. Figure 1 shows schematic illustrations of the MIP coated sensors for traditional mass sensing in liquids (Figure 1a,b) and for additional conductance measurement (Figure 1c,d). 

Temperature-controlled measurements of the yeast cell binding were performed with an oscillator circuit in microfluidic flow chambers in laminar flow conditions. Results are plotted as differential measurements of the serial resonance frequencies of sensitive and reference electrodes on the same quartz crystal.

Compressed *S. cerevisiae* were rehydrated in phosphate buffered saline (PBS) and the concentrations were determined with a Neubauer Improved cell counting chamber. Elevated temperatures of 70 °C were used to prepare suspensions with non-viable cells which cause a slight volume reduction of the yeast. The viability of the yeast cells was validated with methylene blue staining.

## 5. Use Case Microorganism Detection

**Figure 2:**
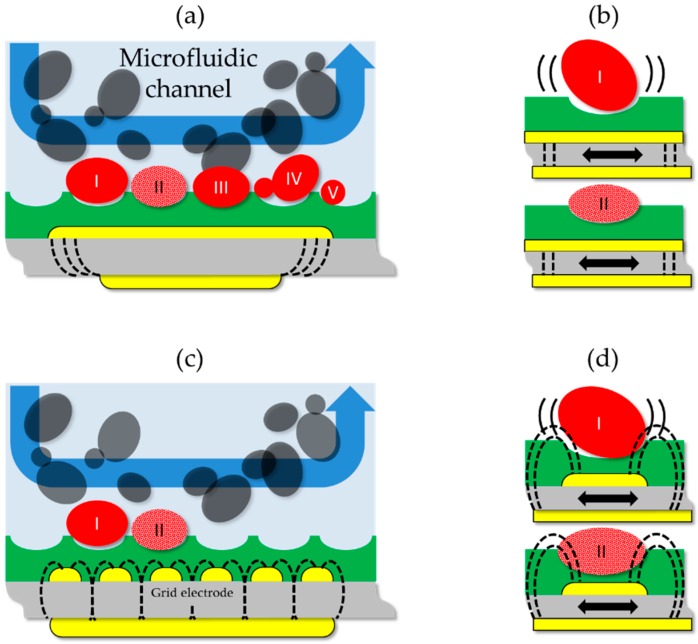
Schematic illustrations of the MIP layer mediated resonant coupling effects between (I) large viable yeast cells, (II) non-viable cells, (III) viable cells matching the mould, (IV) budding yeast cells, and (V) small cells and the transducer. (**a**,**b**) Mass-sensitive effects with conventional electrodes are related to the growth stage of the yeast cells and their viability. The electrode geometry allows excluding capacitive coupling to the medium. Without sufficient geometrical fit, the mass sensitivity for cells larger than the imprint (I) reduces. Non-viable cells with smaller diameter (II) show perfect fit in the moulded pits and ideal mechanical coupling to the MIP layer. (**c**,**d**) The additional fringe fields with grid electrodes allow probing of the dielectric properties of yeast cells and diminishes the gravimetric response.

As mentioned in the previous sections, the chemical sensor is not only the MIP layer but equally important is the right transducer for the right application. For the extreme case of micron-sized cellular analytes, the mechanical coupling element between the analyte and the resonating piezoacoustic transducer mediated by the moulded thin film layer offers a unique opportunity to detect not only specifically cell mass but even cell viability. Sensitive detection of cellular analytes is not a trivial task. Point contacts between a spherical analyte and the transducer surface lead to rather low sensitivity even in cases of a sensor layer from highly affine antibodies or alternative binders, such as nucleotide aptamers, carbohydrates or lectins [16,17,18]. With cells resting on a plane thin film surface, very little mass effects can be measured with a QCM and with decreasing geometrical fit between the mould and analyte, the sensitivity diminishes (Figure 2).

For microorganism measurements in rather harsh conditions, additional constraints for the binder need to be considered, such as media matrix effects, cell population heterogeneity, abrasive flow conditions at high flow rates, sedimentation effects, fluidic drag forces, and biofouling. Furthermore, for multiple or continuous testing, robustness over days is favorable and a high reversibility for the sensor signal is required. Such measurement conditions are obviously not the domain of natural antibodies and most importantly one needs to consider interfering forces for the binding event, such as drag forces as well as aggregates and air bubbles. 

**Figure 3:**
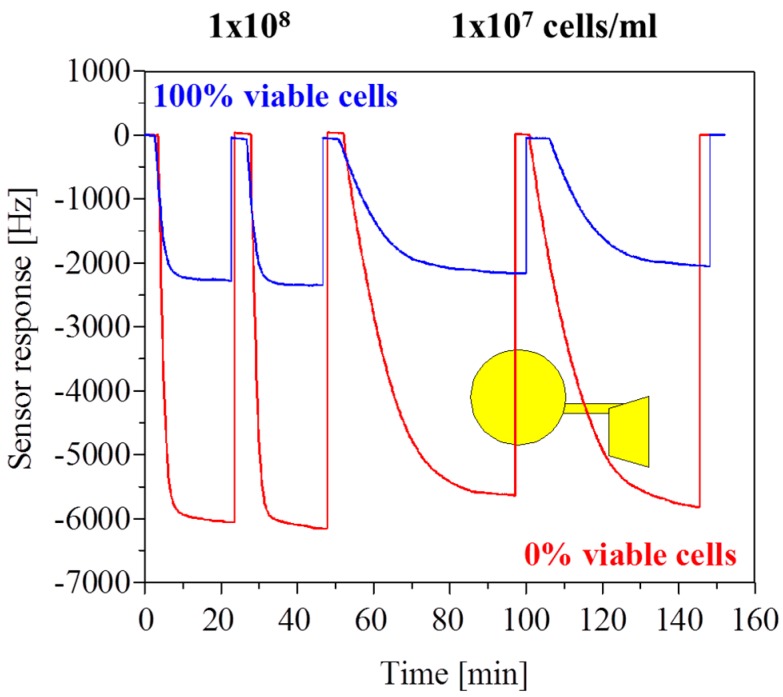
Superimposed differential measurements with a conventional QCM electrode layout for high *S. cerevisiae* suspensions in PBS buffer with 100% viable cells (blue) and non-viable cells (red).

In Figure 3 a bioreactor condition with high yeast concentrations of 10^7^ to 10^8^ cells/mL is simulated and cell concentrations are measured with conventional electrode geometry covered with a *S. cerevisiae* moulded polurethane layer as schematically illustrated in Figure 2b. With the chosen continuous flow conditions of 5 mL/min, 90% of the gravimetric sensor response is achieved within 30 min and the sensor response is fully reversible by a short washing step at high flow rates. The two superimposed differential measurements of sensitive and reference electrodes show the response of the same MIP layer for 100% and non-viable cells. The reference sensor coated with an unmolded thin film shows only a <1% response compared to the sensitive channel (data not shown). Hence, the drag force due to the high flow rate does not allow sedimentation in the flow cell and only weak unspecific adhesion of *S. cerevisiae* occurs which cannot be detected due to point contacts. The almost three-fold enhanced sensor effect with non-viable cell suspensions is related to the slight volume reduction of the non-viable cells after thermal shock which leads to an improved match of the target cells to the compressed yeast cell size used for surface imprinting. Similar results can be obtained with synchronized cell suspensions in different growth stages (Figure 2a), where insufficient geometrical fit of budding yeast or spores causes reduced mechanical coupling and thus sensitivity. Note, the imprints have a depth of 20%–30% of the templating cell diameter which means that cells are not getting “arrested” but rather a dynamic equilibrium is achieved in a continuous flow cell where cells will show an increased mean residence time only with a good fit to the pit. Reduced sensitivity is also observed with increasing vertical orientation of the QCM which indicates low binding affinities as compared to the fluidic forces. However, the chemical recognition is equally important, as has been demonstrated for different *Saccharomyces* species [12], polymer compositions, and matrix effects from growth media [13]. Using templates from synchronized cultures allows optimization of the sensitivity for a respective yeast cell cycle stage [19]. In brief, with the appropriate surface imprinted layer one can solve a complex analytical question targeting a highly variable analyte in even harsh and highly opaque conditions with a simple MIP coated QCM. To quantify the interaction process between cells and the pits, more work is required as probing of the chemistry in, for instance, buffer with variable pH also causes cellular stress and thus may change the binding conditions due to cell swelling.

**Figure 4:**
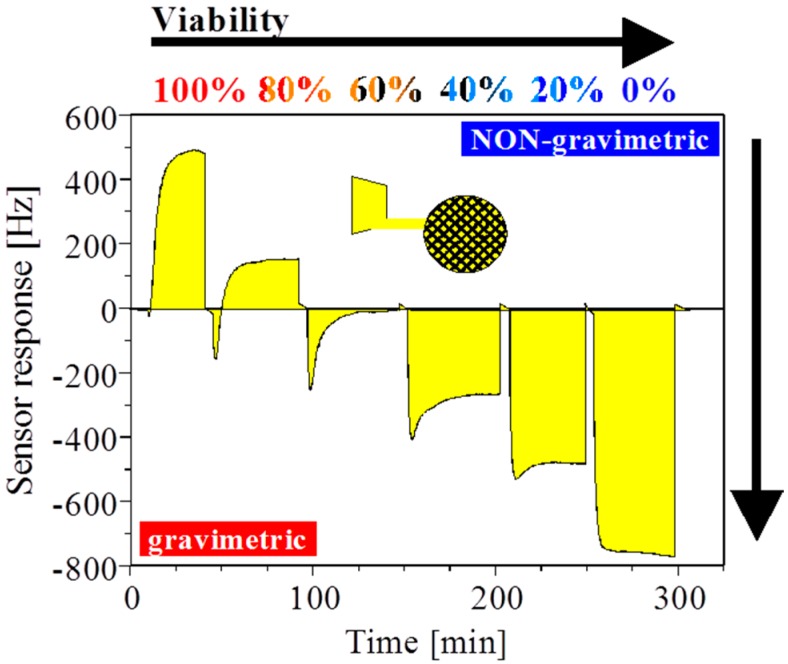
Differential sensor response with a *S. cerevisiae* imprinted layer on a grid electrode. All samples had a constant cell concentration of 10^7^ cells/mL. Gravimetric and non-gravimetric responses are observed depending on the cell viability. The area under the response curve is colored in yellow to guide the eye.

For a fermentation process control sensor, not only is the cell concentration relevant, but equally important is the information on the viability of the cells. Viability of the cell is related to intact plasma and vacuolar membranes which is usually monitored by dielectric spectroscopy [20,21]. For mass-sensitive QCMs with MIP layers, dielectric properties are usually avoided (Figure 2a). However, with a grid-type electrode as schematically illustrated in Figure 2c, the QCM can become very sensitive for ionic effects. The electric field penetrates via the grid into the MIP layer where the pits can be empty or filled with yeast cells. With an appropriately thin polymer layer, the electrical interference from the fringe fields can now be used to probe the viability of cells. Figure 4 shows identical measurement conditions to Figure 3 but the grounded electrode facing the suspension of 10^7^ cells/mL has a grid structure. With this experimental setting we can observe that the massive gravimetric effect cannot be observed anymore for both viable and non-viable yeast cells. The eight-fold diminished sensitivity is the result of the reduced metallized electrode area. Second, the sensor response is inverted and non-gravimetric responses dominate a cell suspension with >60% viability. Viable cells with integral membranes have a high capacitance and the interior conductivity differs from non-viable cells [22]. Third, the shape of the positive frequency shift persists but linearly decreases with decreasing viability (note: cell concentration is constant). Fourth, the total response is dominated by mass-sensitive effects with <60% viable cells. Last, the sensor response with non-viable cells is almost quantitatively gravimetric. In conclusion, we observe two overlapping and independent sensor effects with two comparable time constants at the given continuous flow conditions. 

**Figure 5:**
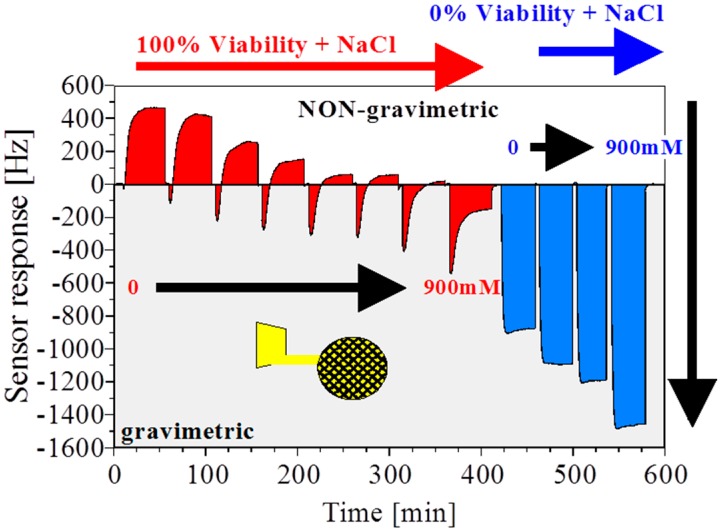
Differential sensor response with a *S. cerevisiae* imprinted layer on a grid electrode with an increasing concentration of NaCl in phosphate-buffered saline (PBS). All samples had a constant cell concentration of 10^7^ cells/mL. Non-gravimetric responses are observed independent on the buffer conductivity. Increasing conductance causes a pseudo-gravimetric response due to capacitive coupling of the medium. The area under the response curve is colored in red for viable and blue for non-viable cells to guide the eye.

To determine the influence of conductivity NaCl is stepwise increased by 100 mM to the PBS buffer and the experiments are repeated with viable and non-viable yeast (Figure 5). Note, *S. cerevisiae* is a highly osmotolerant microorganism and the addition of NaCl does not lead to a significant decrease of the viability but only increases osmotic stress [23]. For the case of 100% viable cell suspension, the positive frequency shift remains constant independent from the increasing conductivity of the buffer. We again can observe two superimposing effects. The fast negative frequency response appears to be related to a lossy capacitance due to the increased ion concentration. The second delayed effect causing positive frequency shifts shows the same time constant as in Figure 4 for 100% viable cells and inverts the fast response to NaCl due to increased permittivity of the bound cells. Finally, adding NaCl to non-viable cell leads to an equally fast response as observed for the viable cells. However, the non-viable cells show low permittivity and thus the conductance gradually increases with increasing NaCl. This leads to the conclusion that medium conductivity effects are not responsible for non-gravimetric frequency increases. It is difficult to experimentally quantify the nature of the observed frequency increase but phase delays between viscoelastic layers on resonators are known to cause such frequency shifts [24]. In the observed case with the grid electrodes and the rigid MIP layer, one can assume that the positive frequency shift is weakly related to mechanically and thus capacitively coupled bound yeast cells to the rigid moulded thin film (cf. Figure 2d). This effect does not appear with a MIP layer on a conventional QCM electrode layout where the dominating sensor effect is always gravimetric, irrespective of the cell viabilities and thus size. In conclusion, with the appropriate strategy for the transducer and the right methodology to detect cells with high sensitivity, surface-imprinted films with low binding affinity can make a significant difference to conventional binders due to the opportunity for mechanical coupling to a resonating device. In addition, depending on the electrode layout, MIP coated QCM can be tuned to screen microorganism mass, growth stage, and viability. A detailed discussion on the chemical recognition effect between cells and MIPs has been reported elsewhere [13].

The key learning of the use case is that the transducer is equally important as the MIP fabrication and with appropriate soft-lithography and electrode design, not only can chemical recognition be achieved but additional cellular information on morphology and viability is also gained. Without moulding of the MIP layer, the mechanical coupling to the resonating device is too low and the sensitivity is lost. Moulded layers can also be fabricated on equally sensitive surface plasmon resonance transducer but the bulky analytes requiring micron-thick thin films can barely be detected in comparable conditions, as discussed for the piezoacoustic devices. Furthermore, a non-specific sedimentation effect cannot be discriminated from specific recognition due to the missing acousto-mechanical probing of the fit between pit and cells.

## 6. Random or Pre-Defined Polymeric Sequence for Synthetic Binder

Although the soft-lithography method for MIP fabrication is broadly applicable, it will always depend on the presence of a biological template to form binding sites. For the commercialization of MIP biosensors such a dependency could be critical as the sensor fabrication will require the preparation of a stamp with a biological template, which again needs to be fabricated. This waste of material and increased complexity along the value chain makes alternative binders with a rather deterministic binding site, such as nucleic acid and peptide aptamers, more attractive for scalable sensor fabrication. The main limitation of most reported MIP sensors is thus the intrinsic dependence on a template during the polymerization and the random sequence which leads to intrinsic binding site heterogeneities. However, binders with a predefined polymeric sequence have their own limitations, such as biodegradability. In addition, polynucleotide binders appear to have a strong dependence from pH and ionic strength which limits broad applicability in harsh conditions [25,26]. Peptide aptamers with a rather deterministic design variability of the binding site embedded in a neutral protein scaffold probably unite the best of both worlds–natural antibody-like affinities and MIP-analogue robust scaffolds [27]. Despite the recent success of aptamers, MIPs are by no means unattractive and for chemical sensing probably still superior due to long-term stability. I believe the community could take more advantage of the unique MIP robustness and adopt techniques from related communities to further develop their art in designing synthetic binders for sensing. Why not integrate a peptide binding motif in a synthetic MIP scaffold and use it for harsh conditions which is not the domain of natural antibodies? Most recently, first attempts on hybrid MIP-DNA aptamer coatings have been reported and it will be interesting to learn if the MIP sensor community will be successful in applying such adopted receptor strategies in the future [28,29]. 

Equally important to the imprinting is the translation of coating methods from the research laboratory to a scalable manufacturing process. Most recently, the group of Piletsky reported on a MIP synthesis method for imprinted nanoparticles in water using an automated chemical reactor [30]. This concept appears attractive for chemical sensing if one can further develop an efficient top-down transducer coating methodology with the bottom-up fabricated MIP nanoparticles. To improve binding affinities for bioanalyte recognition in a size regime which requires surface-imprinting, such as proteins or cells, a similar strategy with a peptide binder could be applied. In this case, one could envision chemical sensing which outperforms a natural antibody by recognizing not only the surface chemistry but even the overall morphology of the bioanalyte [12] or some microorganism functionality [31].

## 7. Conclusions

The robust nature of MIP binders is without any question intriguing for chemical sensing but the market forces are a substantial hurdle to translate template-directed polymerization methods into innovative chemical sensor products. To achieve a successful translation, I believe the community requires more research efforts on a system level which includes application-driven work on both transducer and coatings. The right choice of the transducer is often neglected but critical for a viable product. A famous reference example on the impact of a transducer in biosensing is the market penetration of low-cost electrochemical glucose sensors replacing more and more optical sensors [32]. For small molecule analytes, I believe development readiness has been proven and small arrays have a good chance against physical sensors and miniaturized analytical instrumentation due to the MIP flexibility to customize sensors for a respective application. In the case of biosensing with bulky analytes, soft-lithography is a broadly applicable and rather deterministic methodology for high density receptors on thin film surfaces. To stress the importance of the transducer, I highlighted a scenario with surface-imprinted layers on piezoacoustic sensors which allows both good mechanical coupling of imprinted pits to bulky cellular analytes and complete reversibility. With this approach, cellular viability information is obtained which would otherwise be difficult to achieve with a natural antibody layer on a QCM. Last, the intrinsic dependency on the biological template is a disadvantage for the commercialization of bioimprinted sensors. A combination of both a thin film moulding and robust aptamers could be an interesting combination to further develop synthetic binders for commercially viable chemical sensor products.

## Abbreviations

IVD	in-vitro diagnostics
MIP	molecularly-imprinted polymer
PBS	phosphate-buffered saline
QCM	quartz crystal microbalance
